# Death of Dr. Nottingham

**Published:** 1876-04

**Authors:** 


As we go to press it is with feelings of no ordinary sorrow that we are called upon to announce to our readers the death of Dr. C. B. Nottingham, of Macon. The profession of Georgia will long and deeply mourn the loss of one so useful to the community; one so ornamental to the profession of his lifetime choice; one so respected and beloved by his brethren. We mourn him as a departed friend, long and fondly cherished; whose life beautifully exemplified the graces of meekness and charity; whose indomitable will and energy were always ready at the call of suffering humanity; whose heart was full of brotherly kindness and self-sacrificing generosity; and whose soul was the very shrine of honor and probity.

We hope in our next issue to be able to present a suitable notice of his life and professional services.

